# Breaking the Diagnostic Barrier: DNAJB9 Heat Shock Protein Testing for Fibrillary Glomerulonephritis in Cases With Low Tissue Reserves

**DOI:** 10.7759/cureus.87305

**Published:** 2025-07-04

**Authors:** Khadija Qureshi, Jerry Kenmoe, Arvind Kunadi

**Affiliations:** 1 Internal Medicine, McLaren Hospital, Flint, USA; 2 Internal Medicine, Michigan State University College of Human Medicine, Flint, USA

**Keywords:** fibrillary glomerulonephritis, immunotherapy, mesangial proliferation, proteinuria, renal biopsy, resistant hypertension

## Abstract

Fibrillary glomerulonephritis (FGN) is an uncommon primary glomerular condition with a vague clinical presentation that poses a diagnostic challenge. We present a case of a 73-year-old white female with severe kidney damage, malignant hypertension, and recurring abdominal pain who underwent a series of extensive tests until her renal biopsy showed eosinophilic deposits with mesangial matrix and GBM growth, indicating segmental membranoproliferative characteristics, and global mesangial proliferation of the glomeruli with fibrillary deposits. The DNAJB9 heat-shock protein was present in the tissue sample, supporting the FGN diagnosis. The lack of clear-cut treatment standards makes managing FGN difficult, and the disease has a dismal prognosis with a 50% possibility of developing end-stage renal disease within an average of 10 years post-diagnosis. Improving patient outcomes and halting further decline in renal function depend on early detection and effective care.

## Introduction

Fibrillary glomerulonephritis is a rarely seen primary glomerular disease characterized by Congo red-negative mesangial growth and deposition of fibrillar material [1]. Less than 1% of primary glomerular disorders are caused by this condition [2]. Immunohistochemistry and electron microscopy, which show the distinctive fibrillary deposits, are used to confirm the diagnosis. Hematuria, proteinuria, hypertension, and renal failure are frequently seen in fibrillary glomerulonephritis (FGN) patients [3]. Although there is still much to be studied about the pathophysiology of FGN, certain cases have been linked to underlying autoimmune disorders, infections, and malignancies [4]. One patient may have stable renal function for years, while another develops end-stage renal disease (ESRD) within months as a result of FGN's very varied clinical course. FGN has a poor prognosis and complex therapy with a 50% chance of developing ESRD within 10 years of diagnosis [1]. This report will underpin the case of a 73-year-old woman who experienced chronic abdominal pain, malignant hypertension, and acute renal injury and was eventually diagnosed with FGN. The need to keep a high index of suspicion for FGN in patients with unexplained hypertension and acute renal damage is emphasized in this case.

## Case presentation

A 73-year-old Caucasian female with a known medical history of hyperlipidemia, hypertension, dementia, gastritis, and diverticulosis presented to the emergency room with the main complaints of nausea, vomiting, and abdominal pain for the past three days. The patient had a history of three similar hospitalizations within the last six months, with significant malignant hypertension and acute kidney injury. Upon admission, her vitals were as follows: blood pressure 193/136 mmHg, mean arterial pressure (MAP) 183 mmHg, and heart rate 135 beats per minute (bpm). Electrocardiogram (EKG) showed a heart rate of 125 bpm with no significant ST wave abnormalities. Laboratory results did not show any significant abnormalities except for an elevated creatinine level and low estimated glomerular filtration rate (eGFR) (Table 1).

**Table 1:** Inpatient blood and urine test results.

Tests	Results	Reference range
White blood cells	10.6 k/µL	4.0-11.0 k/µL
Hemoglobin	14.8 g/dL	12.0-16.0 g/dL
Platelets	340 k/µL	150-450 k/µL
Anion gap	15.3 mmol/L	7.0-16.0 mmol/L
Sodium	136 mmol/L	135-145 mmol/L
Potassium	4.0 mmol/L	3.5-5.0 mmol/L
Chloride	101 mmol/L	98-107 mmol/L
Bicarbonate	19.9 mmol/L	22-32 mmol/L
Creatinine	1.5 mg/dL	0.5-1.1 mg/dL
eGFR	35 mL/min/1.73 m²	>90 mL/min/1.73 m²
BUN	12.3 mg/dL	7-20 mg/dL
Alanine transaminase (ALT)	11 U/L	0-45 U/L
Aspartate aminotransferase (AST)	24 U/L	0-40 U/L
Protein (urine)	3+	Negative
Leukocyte esterase (urine)	Negative	Negative
Nitrites (urine)	Negative	Negative
Crystals (urine)	None	None
Hyaline casts (urine)	0-5	None

**Figure 1:**
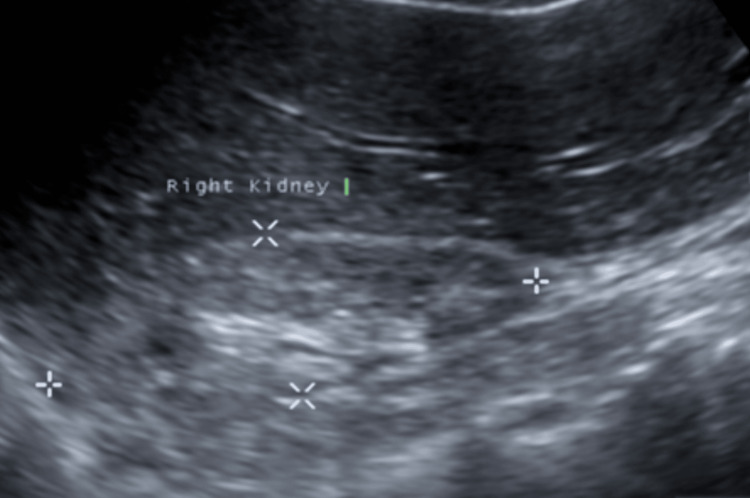
Retroperitoneal ultrasound showing an atrophied right kidney.

The patient was initially admitted with a diagnosis of colitis as her CT abdomen and pelvis without contrast showed mild-to-moderate colitis with diverticular disease of the left colon without any bowel obstruction or perforation. Treatment with intravenous ciprofloxacin and metronidazole was initiated, and the patient's gastrointestinal symptoms responded well; however, her hypertension remained difficult to control, with systolic blood pressure ranging from 180 to 200 mmHg and diastolic blood pressure ranging from 100 to 130 mmHg. Her acute kidney injury persisted, with a baseline creatinine of 1.3-1.4 mg/dL but increased to 1.7-2.1 mg/dL during subsequent hospitalizations. A retroperitoneal ultrasound showed a right kidney measuring 7.7x3.4x3.2 cm with a thin and hyperechoic right renal cortex measuring 4 mm. Left kidney measured 10.1x5.4x4.7 cm with an 8 mm left renal cortex. No hydronephrosis, stone, or mass in either kidney was identified (Figure 1).

Nephrology was consulted and the patient was followed up in an outpatient setting. Significant laboratory findings during outpatient nephrology follow-up included an elevated urine protein-to-creatinine ratio and high urine protein levels. Complement levels (C3 and C4) were within normal limits, and autoimmune markers, including antinuclear antibody (ANA) and antineutrophil cytoplasmic antibody (ANCA), were negative. Serum protein electrophoresis and immunofixation revealed the absence of monoclonal immunoglobulins. The patient had another hospitalization in December 2022, presenting with malignant hypertension (200s/130s mmHg) and acute kidney injury. At a nephrology clinic follow-up, the patient was found to have worsened total urine protein level as well as elevated protein-to-creatinine ratio (Table 2). These findings were indicative of significant proteinuria, reflecting ongoing glomerular injury and progression of kidney damage.

**Table 2:** Subsequent blood and urine workup showing worsening kidney disease and proteinuria.

Tests	Results	Reference ranges
Urine protein (random)	795 mg/dL	<150 mg/dL
Urine protein-to-creatinine ratio	7.430	<0.2
Serum creatinine	2.12 mg/dL	0.6-1.3 mg/dL
Blood urea nitrogen (BUN)	30 mg/dL	7-20 mg/dL
eGFR	24 mL/min/1.73 m²	>60 mL/min/1.73 m²
Urine protein (follow-up)	1148 mg/dL	<150 mg/dL
Protein-to-creatinine ratio (follow-up)	8.367	<0.2

**Figure 2:**
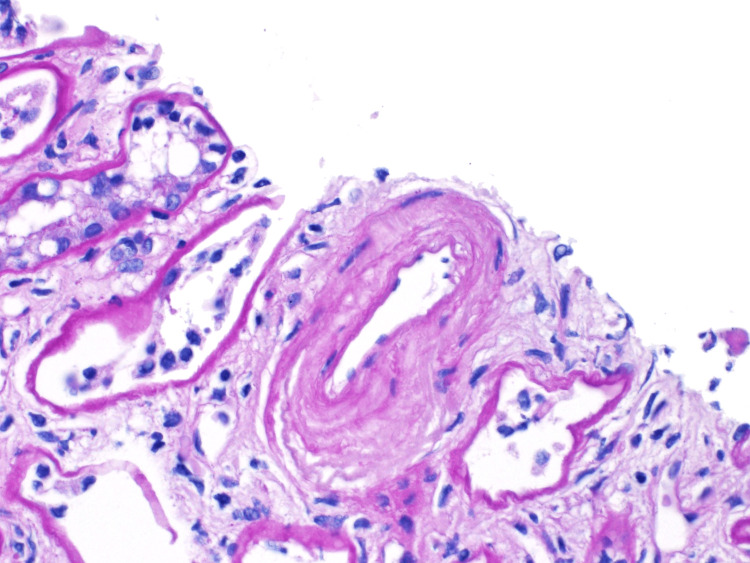
Arterial intimal fibrosis. The histopathology of this renal biopsy slide shows arterial intimal fibrosis with concentric thickening of the intimal layer leading to luminal narrowing. These chronic vascular changes can lead to ischemic injury and progressive nephron loss in the setting of fibrillary glomerulonephritis (FGN).

**Figure 3:**
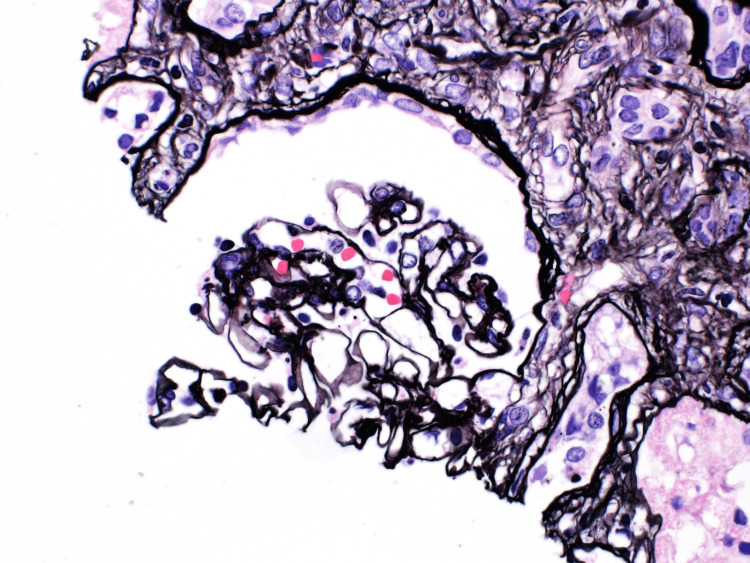
Capillary wrinkling secondary to fibrillary glomerulonephritis (FGN). This renal biopsy slide demonstrates capillary wall wrinkling associated with fibrillary glomerulonephritis (FGN), characterized by collapsed and irregular glomerular capillary loops. This finding reflects chronic glomerular damage, often accompanied by mesangial matrix expansion and deposition of randomly arranged fibrils.

**Figure 4:**
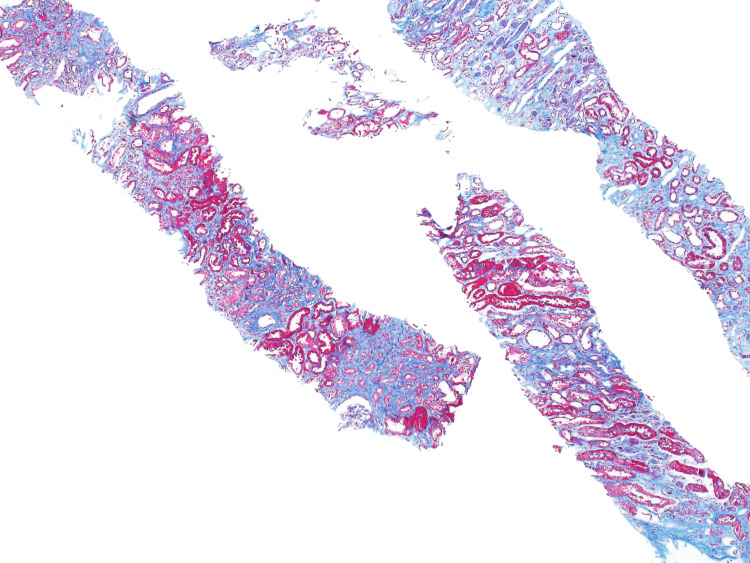
Renal biopsy slide showing interstitial fibrosis supporting the diagnosis of FGN. FGN: fibrillary glomerulonephritis

**Figure 5:**
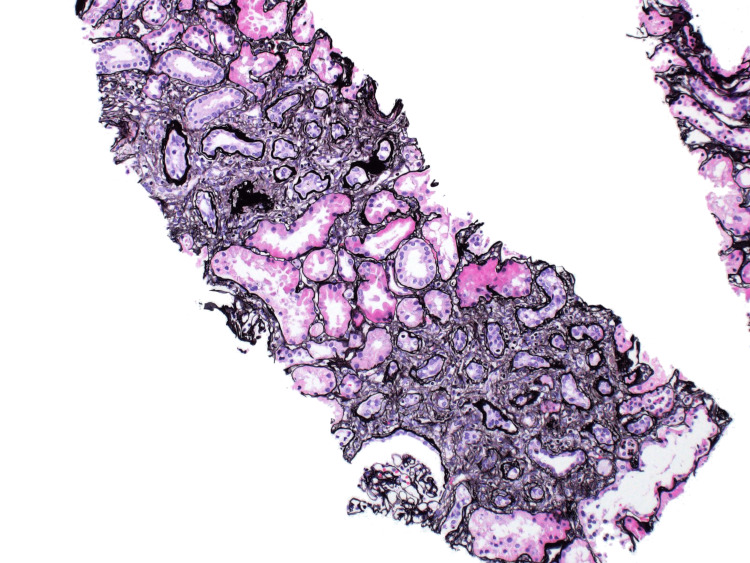
Renal tubular atrophy secondary to fibrosis. This slide demonstrates the distorted architecture of the renal tubules secondary to chronic tubular injury. The histopathology is significant for thickened tubular basement membranes and interstitial fibrosis in the setting of fibrillary glomerulonephritis.

Due to her worsening renal function results, a renal biopsy was performed. The pathology results of the renal biopsy samples revealed global mesangial proliferation of the glomeruli with fibrillary deposits, expansion of the glomerular basement membrane, arterial intimal fibrosis, and chronic vascular changes within the mesangial matrix, demonstrating segmental membranoproliferative features (Figures 2, 3). The biopsy slides also showed interstitial fibrosis and renal tubular atrophy (Figures 4, 5).

**Figure 6:**
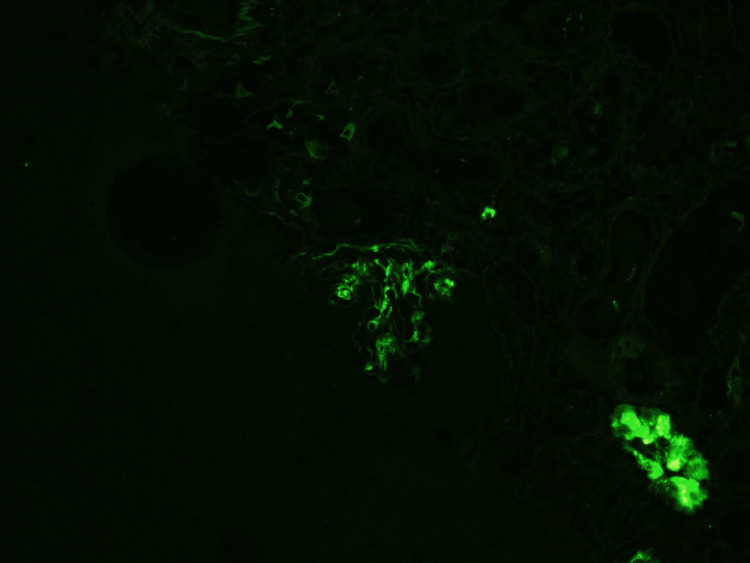
Immunofluorescence depicting IgG deposits in the glomerular membrane. Immunofluorescence microscopy in fibrillary glomerulonephritis (FGN) shows coarse polyclonal granular IgG deposits along the glomerular capillary walls and mesangium without a linear or peripheral pattern, which helps distinguish FGN from other conditions like anti-GBM disease. Additionally, in FGN, the fibrils are composed mainly of IgG immunoglobulins, not amyloid proteins. The IgG deposits are slightly thicker (typically 16-24 nm) than amyloid fibrils. The absence of staining for Congo red (in most cases) and the composition of the fibrils help to differentiate FGN from amyloidosis.

**Figure 7:**
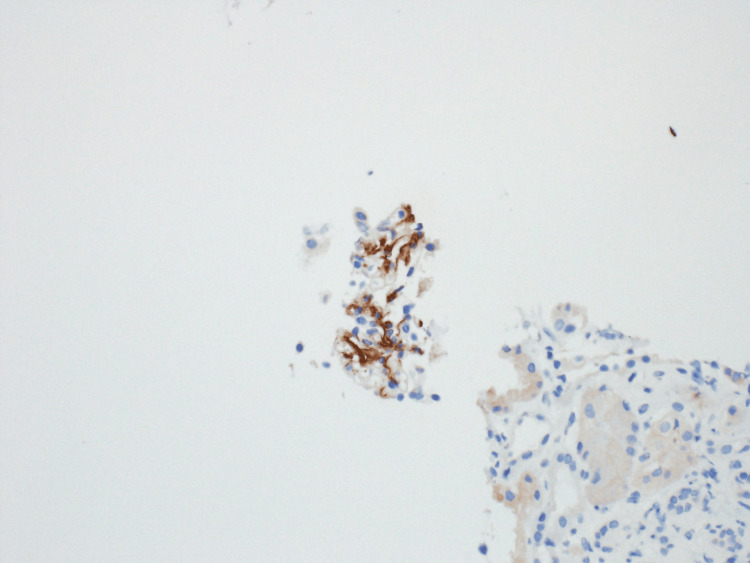
Immunohistochemistry showing DNAJB9 protein biomarker. This figure shows strong extracellular staining of DNAJB9 protein within the glomeruli, highlighting the presence of abnormal fibrillary deposits. DNAJB9 is a highly specific protein biomarker for fibrillary glomerulonephritis (FGN).

Immunofluorescence performed on the biopsy sample was significant for IgG deposits in the glomerular membrane, which were negative for Congo Red staining (Figure 6). The presence of IgG deposits on renal biopsy also helped rule out hypertensive nephrosclerosis, which typically lacks significant immune complex deposition. However, with low tissue sample availability from the biopsy, immunohistochemical staining for DNAJB9 protein was ordered and returned positive, affirming the diagnosis (Figure 7).

## Discussion

Fibrillary glomerulonephritis (FGN) is an unusual and poorly understood glomerular disease, posing challenges in accurate diagnosis secondary to its vague clinical presentation and low prevalence. The incidence of FGN is estimated to be less than 1% of all primary glomerular diseases and is more common in older adults, with a slight female predominance [5]. The exact etiology and pathogenesis of FGN have yet to be well understood, but the disease is associated with fibrillary protein accumulation in the glomeruli. Fibrillary deposits that stain positively with Congo red are a hallmark of FGN, but unlike amyloidosis, they do not exhibit the usual apple-green birefringence under polarized light [6]. A varied array of clinical symptoms, such as proteinuria, hematuria, edema, hypertension, and renal insufficiency, can be present in FGN and mimic those of other primary glomerular disorders [7].

As highlighted in the case presentation, our patient had recurrent vomiting, abdominal pain, and diarrhea with a medical history notable for diverticulosis, gastritis, hyperlipidemia, and hypertension. The patient's hospitalization was significant for the emergence of colitis, acute kidney injury, and malignant hypertension. Symptoms were initially investigated to rule out inflammatory and viral causes. However, further testing, which included a kidney biopsy, was necessary given the patient's repeated hospitalizations and deteriorating renal function. The renal biopsy revealed eosinophilic deposits involving the mesangial matrix and extending into the glomerular basement membrane (GBM), global mesangial proliferation with fibrillary deposits, and expansion of the GBM - findings suggestive of membranoproliferative glomerulonephritis (MPGN) pattern, characteristic of fibrillary glomerulonephritis (FGN). The DNAJB9 heat-shock protein was present in the tissue sample, supporting the FGN diagnosis.

The DNAJB9 protein, also known as Mdg-1 or ERdj4, operates as a cochaperone of heat-shock protein 70, which is involved in endoplasmic reticulum protein folding mechanisms. It modulates the ATPase activity of 70 kDa heat shock proteins as a member of the DNAJ heat shock protein family (Hsp40), impacting different cellular functions [8]. DNAJB9 is a 223-amino-acid protein abundant in secretory organs and participates in the unfolded protein response (UPR). It has been linked to endoplasmic reticulum stress and the UPR, and it is a heat shock protein cochaperone, regulating various cellular functions [9]. Although no circulating anti-DNAJB9 antibodies have been detected, the presence of full-length DNAJB9 protein in immunological deposits and the absence of DNAJB9 mutations in examined patients suggest that DNAJB9 may be the originator of FGN. Another hypothesis is that DNAJB9 binds to misfolded IgG, supported by its known biological functions and proclivity to bind aggregation-prone peptide sequences [8,9]. The precise role of DNAJB9 in FGN is unknown, and ongoing research aims to shed light on this.

The practical superiority of DNAJB9 in FGN was illustrated in a recent report from the Mayo Clinic. The clinical data from 18 patients with renal biopsy findings consistent with FGN were studied. The glomerular deposits showed apple-green birefringence when stained with Congo red dye and viewed under polarized light. This "Congophilia" is the hallmark of amyloidosis, often used to exclude the diagnosis of FGN. However, the immunohistochemical staining of these samples revealed abundant expression of DNAJB9 and the absence of a proteomic signature of amyloidosis, thus aiding the diagnosis of FGN. In addition, a positive test for DNAJB9 markers can still yield a reliable diagnosis even in low tissue reserves or inadequate tissue biopsy samples [10].

Interestingly, extracellular DNAJB9 protein, abundantly present in the glomeruli of 98% of FGN cases, has emerged as a specific immunohistochemical marker for fibrillary glomerulonephritis (FGN). The diagnosis of FGN has been completely transformed by this finding, which has rendered DNAJB9 as the new gold standard and eliminated the need for excessive reliance on electron microscopy [11]. The cases of "Congophilic fibrillary glomerulonephritis" can be diagnosed with DNAJB9, which formerly could have been misdiagnosed as amyloidosis, with significant treatment implications. Due to the lack of clear-cut treatment modalities, managing FGN is difficult. Treatment aimed to reduce proteinuria, hypertension, and edema while halting future renal function deterioration [12]. Immunosuppressive medications such as prednisolone, cyclophosphamide, mycophenolate mofetil, and rituximab are available treatments for FGN [13]. More information is needed to determine the effectiveness of various treatments. A 50% chance of developing end-stage renal disease within 10 years of diagnosis is another factor contributing to the disease's poor prognosis [9]. The level of proteinuria at the time of diagnosis is the strongest indicator of renal survival, with individuals with proteinuria in the nephrotic range having a worse prognosis [14]. As fibrillary glomerulonephritis is a rare primary glomerular disease, the diagnosis can be tricky because of its variable clinical presentation and low prevalence. Improving patient outcomes and halting future decline in renal function depend on early detection and effective care. There is a need for more research into the pathogenesis and therapy of FGN because it is challenging to manage and has few treatment choices [15].

## Conclusions

In conclusion, this report focuses on fibrillary glomerulonephritis (FGN), an uncommon and frequently misdiagnosed primary glomerular illness, and its diagnostic difficulties and clinical ramifications. In order to stop the progressive decline of renal function and to improve patient outcomes, it is essential to identify and treat FGN early. It is crucial to maintain a high index of suspicion for FGN in patients who present with unexplained hypertension and acute renal damage, especially after other plausible alternative diagnoses have been ruled out. The renal biopsy is critical for diagnosis due to its specific histopathological features and immunohistochemical staining for the DNAJB9 protein. Because there are no specific treatment guidelines for FGN, the care plan should be tailored to the patient's clinical presentation and comorbidities. Further research is required to better understand the etiology of FGN and develop targeted therapy options for an improved prognosis for patients with this unusual ailment.
